# Effects of early exercise training on the severity of autonomic dysreflexia following incomplete spinal cord injury in rodents

**DOI:** 10.14814/phy2.14969

**Published:** 2021-08-02

**Authors:** Kathryn A. Harman, Kathryn M. DeVeau, Jordan W. Squair, Christopher R. West, Andrei V. Krassioukov, David S. K. Magnuson

**Affiliations:** ^1^ Department of Health & Sport Sciences University of Louisville Louisville KY USA; ^2^ Kentucky Spinal Cord Injury Research Center University of Louisville Louisville KY USA; ^3^ Department of Anatomy and Cell Biology George Washington University Washington D.C. USA; ^4^ International Collaboration on Repair Discoveries University of British Columbia Vancouver BC Canada; ^5^ Department of Neurological Surgery University of Louisville Louisville KY USA; ^6^ GF Strong Rehabilitation Centre Vancouver Health Authority Vancouver Canada

**Keywords:** autonomic dysreflexia, cardiovascular, exercise, spinal cord injury

## Abstract

Hemodynamic instability and cardiovascular (CV) dysfunction are hallmarks of patients living with cervical and high thoracic spinal cord injuries (SCI). Individuals experience bouts of autonomic dysreflexia (AD) and persistent hypotension which hamper the activities of daily living. Despite the widespread use of exercise training to improve health and CV function after SCI, little is known about how different training modalities impact hemodynamic stability and severity of AD in a model of incomplete SCI. In this study, we used implantable telemetry devices to assess animals with T2 contusions following 3.5 weeks of exercise training initiated 8 days post‐injury: passive hindlimb cycling (T2‐CYC, *n* = 5) or active forelimb swimming (T2‐SW, *n* = 6). Uninjured and non‐exercised SCI control groups were also included (CON, *n* = 6; T2‐CON, *n* = 7; T10‐CON, *n* = 6). Five weeks post‐injury, both T2‐CON and T2‐CYC presented with resting hypotension compared to uninjured CON and T10‐CON groups; no differences were noted in resting blood pressure in T2‐SW versus CON and T10‐CON. Furthermore, pressor responses to colorectal distention (AD) were larger in all T2‐injured groups compared to T10‐CON, and were not attenuated by either form of exercise training. Interestingly, when T2‐injured animals were re‐stratified based on terminal BBB scores (regardless of training group), animals with limited hindlimb recovery (T2‐LOW, *n* = 7) had more severe AD responses. Our results suggest that the spontaneous recovery of locomotor and autonomic function after severe but incomplete T2 SCI also influences the severity of AD, and that short periods (3.5 weeks) of passive hindlimb cycling or active forelimb swimming are ineffective in this model.

## Graphical abstract

Using a high‐thoracic, severe but incomplete model of spinal cord injury we found that passive cycling and swimming, initiated early post‐injury, do not significantly alter the severity of induced autonomic dysreflexia. We discovered that animals with the greatest recovery of hindlimb locomotor function had the smallest pressor responses during induced autonomic dysreflexia.

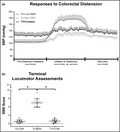

## 1 INTRODUCTION

Individuals with severe spinal cord injuries (SCI) above the T6 spinal level often experience episodic hypertensive crises known as autonomic dysreflexia (AD; Weaver et al., 2012). While the mechanisms underlying AD are likely multifactorial, injury‐induced disruption of supraspinal input onto sympathetic preganglionic neurons in the thoracic spinal cord, increased sensitivity to norepinephrine and/or impaired neuronal reuptake of adrenergic neurotransmitters, and plasticity within the lumbosacral spinal cord are known to be contributing factors (Arnold et al., 1995; Furlan et al., 2003; Hou et al., 2008, 2009). Autonomic dysreflexia is most often triggered by noxious input from peripheral or visceral sources below the injury, including bowel or bladder, which results in extreme vasoconstriction below the injury in addition to upper body flushing, sweating, headache, and reflex‐mediated bradycardia (Cragg & Krassioukov, 2012; Guttmann & Whitteridge, 1947; Karlsson, 1999; Krum et al., 1989). Although uncommon, severe AD can result in life‐threatening events such as cerebral hemorrhage, seizures, and myocardial infarction (Colachis & Clinchot, 1997; Dolinak & Balraj, 2007; Eltorai et al., 1992; Kursh et al., 1977; Lindan et al., 1980; Pine et al., 1991). Importantly, CV consequences of SCI often prove to be a large factor hindering activity‐based rehabilitation programs and exercise participation for SCI individuals (Ashley et al., 1993; Illman et al., 2000).

Severe SCI results in an abrupt immobility that often leads to a chronic sedentary lifestyle with the accompanying cardiovascular (CV) deconditioning and declines in physical fitness and overall health (Groot et al., 2006; Haisma et al., 2006, 2008; Kessler et al., 1986; Thijssen et al., 2006). Unfortunately, implementation of activity‐based rehabilitation is often delayed for months or even years, despite the many studies that have highlighted the benefits of exercise training following injury (Groot et al., 2003; Jacobs et al., 2001; Thijssen et al., 2006). Given that the decline in many facets of physical fitness following SCI is progressive (Thijssen et al., 2006, 2012), the ideal time to initiate exercise and rehabilitation techniques may be early post‐injury prior to the development of significant CV dysfunction. It has been well established in the rat model of complete SCI that anatomical changes of the sympathetic preganglionic neurons occurs between 1 and 14 days post‐injury, and sprouting of CGRP + fibers in the lumbosacral cord is apparent soon after (Beattie et al., 1993; Krassioukov & Weaver, 1995). Modifications to autonomic pathways and the rearborization of sympathetic and caudal circuits seem to be related to both the appearance and severity of AD (Hou et al., 2008; Krassioukov & Weaver, 1996; Lujan et al., 2014; West et al., 2015a). It is already known that appropriately timed exercise training can promote the reorganization of circuits important for locomotor recovery and for the reduction/prevention of neuropathic pain (Houle & Cote, 2013; Nees et al., 2016). Therefore, it is reasonable to speculate that exercise and rehabilitation techniques initiated during the perceived window of opportunity for adaptive plasticity within the spinal cord could also be beneficial for CV recovery and function.

Whereas few clinical studies have attempted to implement exercise programs or activity‐based rehabilitation during the acute and sub‐acute post‐injury phases, there have been some notable animal studies that have examined the effects of various forms of exercise training early post‐SCI (Laird et al., 2009; West et al., 2015b; West, Crawford, et al., 2014). Importantly, West and colleagues showed that passive hindlimb cycling (PHLC) implemented 6 days post‐T3 transection resulted in dramatically improved cardiac function, attenuated AD responses (evoked via colorectal distension, CRD), and reductions in cardiovascular disease (CVD) risk factors (West, Crawford, et al., 2014, 2015). While these studies provided critical proof‐of‐principle insight, most clinical injuries are anatomically incomplete and the degree to which spared circuitry can contribute to proper CV function following acute exercise‐induced plasticity of CV circuits remains poorly understood. Furthermore, it is important to note that the exercise modality employed may differentially affect CV outcomes, as many groups have shown that upper limb exercise alone cannot attenuate the cardiac decline and dysfunction noted in the clinic (Davis et al., 1987; Gates et al., 2002). The only study to date that directly compared active versus passive forms of acutely implemented exercise training following experimental contusion showed that PHLC, but not active forelimb swimming, had the capacity to positively impact flow‐derived indices of cardiac function (DeVeau et al., 2017). This study, however, did not investigate the impact of either exercise training modality on hemodynamic control or severity of AD.

The aim of the present study was to compare two exercise training strategies and their capacity to improve resting hemodynamic parameters and attenuate pressor responses to colorectal distension (i.e., AD) when initiated early after experimental SCI. Active forelimb swimming exercise or PHLC rehabilitation was implemented 8 days following severe, incomplete T2 contusions. After 3.5 weeks of training, animals were instrumented with telemetry devices to assess hemodynamic control and AD severity. The study included uninjured and T10 contused control groups that received no exercise training. As PHLC has been shown to alter the afferent input to the lumbosacral spinal cord and thereby induce positive plasticity in afferents responsible for initiating the AD cascade, we hypothesized that early PHLC training would positively influence CV control and mitigate AD responses to CRD, whereas active forelimb swimming would have no such effect.

## 2 METHODS

### 2.1 Ethical approval and experimental design

All animal care and surgical procedures were performed in accordance with the Canadian Council for Animal Care. Ethics approval was granted by the University of British Columbia.

All experiments were conducted on adult, male Wistar rats weighing 250–300 grams (Harlan Laboratories). Rats were socially housed and maintained on a reverse 12‐hour day/night schedule. Animals were initially divided into three groups: non‐exercised uninjured control (CON, *n* = 6), non‐exercised T10 contusion SCI (T10‐CON, *n* = 6), or T2 contusion SCI (*n* = 20). The T10‐CON group was included to serve as “autonomic dysreflexia controls” for it is generally believed that animals with lesions below the T5 spinal segment typically retain sufficient supraspinal control over the heart and a large portion of sympathetically innervated vascular beds to limit overt CV dysfunction (i.e., AD; Teasell et al., 2000). The addition of this group, therefore, allowed us to examine the impact of lesion level on CV homeostasis irrespective of hindlimb mobility. It is important to note that intact control animals (CON) cannot be subjected to AD provocation. Following SCI procedures, T2 contusion animals were then randomly assigned to one of the following experimental cohorts: non‐exercised T2 SCI (T2‐CON, *n* = 8), T2 SCI plus passive hindlimb cycling (T2‐CYC, *n* = 6), or T2 SCI plus active swimming exercise (T2‐SW, *n* = 6). Exercise strategies were initiated 8 days post‐SCI and lasted for 3.5 weeks. At the termination of the study, all animals were instrumented with telemetry devices to measure blood pressure. Two animals did not survive the implantation, leaving the following group sizes for terminal hemodynamic assessment: CON, *n* = 6; T10‐CON, *n* = 6; T2‐CON, *n* = 7; T2‐CYC, *n* = 5; and T2‐SW, *n* = 6. Following implantation, rats were allowed to recover for 90 min, after which they were assessed for resting hemodynamic control and pressor responses to experimentally induced AD (via colorectal distension, CRD).

### 2.2 Spinal cord injury

Following acclimation to the researchers and testing/exercise equipment, animals received severe 400 kD contusion injuries with a 5 s dwell time using the Infinite Horizons device (IH, Precision Systems & Instrumentation [PSI], Lexington, KY). Previous studies in our lab have shown that this injury model results in approximately 3%–4% sparing of sympathetic axons, making this injury ideal for examining CV dysfunction in rodents (Squair et al., 2016). Importantly, this model produces CV changes that mimic the clinical reality of high‐thoracic and cervical SCI. Rats were anesthetized with isoflurane (5% in induction chamber and maintenance with 2.5%, 1.52 L/min oxygen flow), and administered buprenorphine (0.02 mg kg^−1^, s.c.) and warmed lactated ringers (5 ml, s.c). They were given enrofloxacin (Baytril; 10 mg kg^−1^, s.c., AVP) 3 days prior to and on the day of surgery. A dorsal midline incision was made in the skin and superficial muscle overlying the C7–T3 or T8–T11 vertebrae. A single level laminectomy was performed at the T2 or T9 vertebral level. Following impact, the muscle and skin were closed in layers and rats were given an additional bolus of lactated ringers (5 ml, s.c.) and allowed to recover in a temperature‐controlled environment (33°C, Animal Intensive Care Unit, HotSpot for Birds, Los Angeles, CA). Postoperative care consisted of daily injections of enrofloxacin (10 mg kg^−1^, s.c.), twice‐daily injections of buprenorphine (0.02 mg kg^−1^, s.c.), and twice‐daily 5 ml boluses of lactated ringers for 3 days following surgery. Manual bladder expression was conducted three to four times per day until reflexive voiding was re‐established. Rats were weighed and monitored daily for 2 weeks following SCI, and three times per week thereafter. They were socially housed unless prohibited by aggressive behavior and provided with an enriched standardized diet as previously described (Ramsey et al., 2010).

### 2.3 Rehabilitation interventions

The Basso, Beattie, and Bresnahan Open Field Assessment for Hindlimb Function (BBB) was performed weekly to track locomotor recovery (Basso et al., 1995). For exercised animals, locomotor assessments were conducted in the morning (during the active, “night” part of their schedule), prior to exercise training.

#### 2.3.1 Passive hindlimb cycling

Eight days following contusions, T2‐CYC animals began PHLC training. Passive hindlimb cycling lasted for 30 min each day, 5 days a week for 3.5 weeks (Monday through Friday) using a customized cycle ergometer. Passive hindlimb cycling training has been used extensively in SCI research and details are available elsewhere (Houle et al., 1999; West, Crawford, et al., 2014). Briefly, rats were horizontally suspended on a leather sling with two openings for their hindlimbs. Their hind paws were secured to the pedals with parafilm and gauze padding to minimize skin abrasions. Animals were cycled at a frequency of 0.5 Hz in accordance with previous studies (West, Crawford, et al., 2015). Cereal treats were given to rats during the cycling to encourage compliance. During the final exercise week of the study, two animals developed small sores on their hindlimbs due to the cycling apparatus. These sores were treated with polysporin ointment and extra care was taken when securing their paws to the pedals. In both instances, the sores resolved within 2 days and did not impact exercise adherence. Neither of these animals exhibited typical pain or stress responses (porphyrin around the eyes and nose, reduced exploratory behaviors, biting, etc.), nor did they vocalize/react when their limbs were wrapped in gauze and placed in the pedals during the cycling sessions.

#### 2.3.2 Active swimming

Swimming has been used as both a rehabilitation strategy and as an assessment tool for locomotor recovery following SCI in rodents (Gonzenbach et al., 2012; Smith, Shum‐Siu, et al., 2006). Eight days after injury, T2‐SW animals were re‐introduced to the swimming pool. Animals completed six, 5‐minute swim sessions a day, 5 days per week for 3.5 weeks (Monday through Friday, 30 min total each day). Each swim session consisted of assisted lap swimming (either by tail or trunk support) where the rat was repeatedly placed at one end of a 5‐ft long plexiglass pool and encouraged to swim to the opposite end where they exited the water via a padded ramp. Pool temperatures were maintained at 33–35°C. None of the animals recovered hindlimb kicking; as such swimming following severe contusions is a forelimb‐focused exercise strategy (Smith, Burke, et al., 2006).

### 2.4 Blood pressure assessment

Five weeks following injury, animals were instrumented with blood pressure telemeters and assessed for hemodynamic control during rest and in response to CRD (i.e., AD). Carotid cannulation of the pressure sensor and implantation of the telemetry device were performed under isoflurane anesthetic (5% in induction chamber and maintenance with 2.5%, 1.5–2 L/min oxygen flow). A 3 cm incision was made in the skin between the shoulder blades. Using blunt‐tipped dissection scissors, a small subcutaneous pocket was created to hold the body of the device (model TRM54P, Millar Inc.). The pressure‐sensing catheter tip was tunneled subcutaneously around the neck and exteriorized along the ventral midline. The rostral segment of the carotid artery was occluded and the pressure sensing catheter was inserted and secured with a silk suture. The skin was closed using prolene sutures and rats were administered with an additional bolus of lactated ringers (5 ml, s.c.). As before, animals recovered in a temperature‐controlled environment (33°C, Animal Intensive Care Unit, HotSpot for Birds) for 90 min, after which they were moved to the testing environment for acclimation. Following acclimation in the testing facility (30 min), baseline hemodynamics were acquired for 10 min on awake, freely moving animals. Heart rate was derived from the beat‐to‐beat arterial blood pressure (BP) recording using LabChart, version 8.0 (ADInstruments). Autonomic dysreflexia was assessed in freely moving, injured animals during three clean responses to CRD, a procedure that is routine in the laboratory (Alan et al., 2010; Ramer et al., 2012; West, Crawford, et al., 2015). To evoke CRD, a small plastic balloon was rectally inserted at a distance of 1.5 cm (balloon‐tip of a Swan‐Ganz catheter; 10 mm in length). The balloon was inflated with 2 ml of air over 10 s and distension was maintained for 1 min. Sequential bouts of CRD were performed with at least 10 min in between sessions to allow hemodynamics to return to baseline values. Raw, unfiltered beat‐to‐beat BP and HR data were averaged over 1‐second intervals for each CRD trial. Baseline (pre‐distension, 1 min) hemodynamic parameters were averaged for each session to determine resting BP and HR. Absolute change in systolic blood pressure (SBP), maximum SBP value, and percent SBP increase from baseline were determined for each animal and experimental group averages were calculated for statistical analysis. The time needed to return to resting levels (in seconds), from the point of deflation, was also determined for each session and averaged for each animal/experimental group. To calculate recovery times, second‐by‐second hemodynamic data were assessed during the recovery period and compared to pre‐distension baseline. Time to recovery was calculated using a custom‐made macro that determined the time for hemodynamic measurements to recover within 5 mmHg (SBP; diastolic blood pressure, DBP; and mean arterial pressure, MAP) or 10 bpm (HR) of pre‐distension values for at least 10 consecutive seconds. The time to total recovery (recovery of all four variables) was also determined and averaged for each animal/group.

### 2.5 Statistical analyses

The BBB data were analyzed using nonparametric procedures due to violations of the assumptions of normality and equality of variance necessary for parametric analysis of variance statistical procedures (Hays, 1981). Nonparametric comparisons of independent groups (T10‐CON, T2‐CON, T2‐CYC, and T2‐SW) were performed using Mann–Whitney *U* tests. The weekly timepoints of scores over time within subjects are non‐independent paired data. These were analyzed using Wilcoxon Signed Ranks tests for matched paired data (Seigel, 1988). The left and right hindlimb scores for each animal were averaged after determining there was no significant difference between them. Independent groups were analyzed for measures of CV performance (resting hemodynamics and CV responses to CRD) using nonparametric Mann–Whitney *U* tests. Initial statistical comparisons were made with the original experimental groups (T10‐CON, T2‐CON, T2‐CYC, and T2‐SW) and then animals were re‐analyzed based on terminal hindlimb open‐field performance. For this subsequent analyses, all animals with T2 contusion, regardless of original treatment condition, were stratified according to terminal (week 5) BBB scores: Low BBB <6 (T2‐LOW) or Moderate BBB ≥6 (T2‐MOD) and compared to T10‐CON. This new grouping was made because terminal BBB data exhibited a bimodal distribution––all of the BBB scores ranged from either 0–2 or from 6–11 (representing 58 and 42%, respectively). Subsets of all T2‐SW, T2‐CYC, and T2‐CON groups resided in both LOW (.81 ± .59, mean ± SD) and MOD (7.45 ± 1.6, mean ± SD) BBB categories. The T10‐CON animals made up the third group, all of whom had BBBs between 0 and 2 (.92 ± .66, mean ± SD). These animals represented a unique third group based on injury level due to the distinct CV response differences between T2 and T10 injury models. BBB scores of less than 6 indicate slight or extensive movement of one or two hindlimb joints only, whereas animals with scores equal to or greater than 6 had movement of all three hindlimb joints, with at least extensive movement of two of those joints. With this stratification, animals that had not regained extensive movements of at least two hindlimb joints were considered to be “low” performers. Conversely, animals placed in the T2‐MOD group routinely displayed extensive movement of the ankle, knee, and hip joints and some animals showed plantar placement of the hind paws with or without weight support in stance. Following this re‐stratification, data were analyzed using the same statistical procedures used for the original experimental groups. Statistical analyses were performed with SPSS (v22, Chicago, IL) and statistical significance was set at *p* ≤ .05. Data are presented in the text as means ± standard deviation (SD) and represented graphically using means ± standard error (SEM).

## 3 RESULTS

### 3.1 Spontaneous recovery of hindlimb locomotor function

**FIGURE 1:**
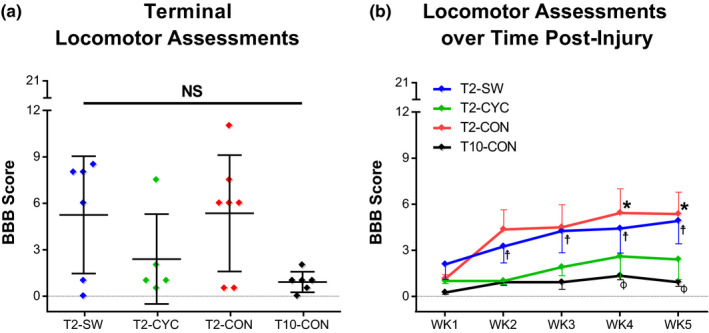
Animals with severe contusion injuries are unable to step with their hindlimbs. (a) Terminal performance on the BBB hindlimb locomotor scale. No differences were noted between groups. (b) Improvements in group BBB scores over time following T2 or T10 contusion as compared to week 1 scores. Data are displayed as mean ± SEM. Significance was set as: T2‐CON, *p ≤ .05 versus week 1; T2‐SW, †*p* ≤ .05 versus week 1; and T10‐CON, ϕp ≤ .05 versus week 1

For T2‐SW and T2‐CYC animals, BBB assessments were performed prior to their daily exercise training. Most animals remained in the early stage of locomotor recovery in which there was no weight support of the hindlimbs (BBB <8), regardless of training group. There were no significant group differences at the terminal time point and variability in BBB scores was high for the T2‐SW, T2‐CYC, and T2‐CON groups (Figure 1a). All T2 groups performed better on the BBB than T10‐CON 1 week after injury (data not shown; *p* ≤ .05 for all comparisons), and T2‐SW animals continued to have better hindlimb function at weeks 2 and 3 versus T10‐CON (data not shown; *p* ≤ .05). Timewise comparisons showed significant hindlimb improvements over time in T2‐SW, T2‐CON, and T10‐CON (Figure 1b; T2‐SW: week 1 vs. weeks 2, 3, 4, and 5, *p* ≤ .05; T2‐CON: week 1 vs. weeks 4 and 5, *p* ≤ .05; T10‐CON: week 1 vs. weeks 4 and 5, *p* ≤ .05).

### 3.2 Swimming improves resting hemodynamic parameters following T2 SCI

**FIGURE 2:**
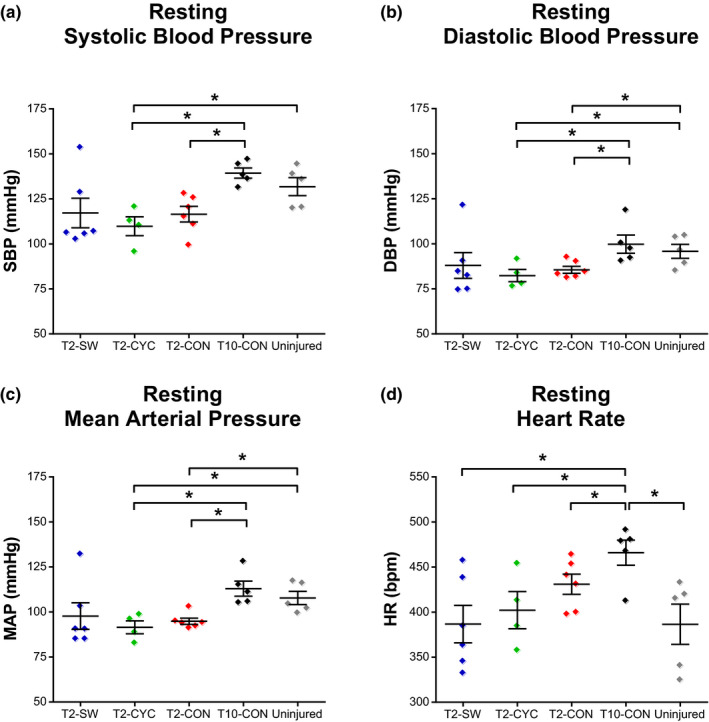
Swimming exercise partially restores resting hemodynamic control following severe contusion to the upper thoracic spinal cord. Resting hemodynamic parameters were measured prior to the CRD protocol 5 weeks post‐SCI. Due to transmitter malfunction, four animals were removed from the analysis. T2‐CON and T2‐CYC animals exhibited significantly reduced systolic (a), diastolic (b), and mean (c) blood pressure as compared to T10‐CON and uninjured control animals. No differences were noted in hemodynamic values between T2‐SW and T10‐CON and uninjured control animals. Severe T10 contusion caused resting tachycardia that was significantly greater than all other groups (d). Data are displayed as mean ± SEM and significance was set at *p ≤ .05

Hemodynamic measures were acquired prior to CRD for all SCI conditions and compared to uninjured controls (CON) at rest. Severe contusion at T10 resulted in resting tachycardia compared to uninjured CON and all T2‐injured animals, regardless of exercise intervention (Figure 2d; T10‐CON vs. CON, *p* = .047; vs. T2‐SW, *p* = .018; vs. T2‐CYC, *p* = .05; vs. T2‐CON, *p* = .045). No differences were noted in SBP, DBP, or MAP between uninjured CON and T10‐CON animals. Severe contusion at T2 resulted in low resting blood pressure values compared to both uninjured CON and T10‐CON. However, swimming exercise (T2‐SW) appeared to normalize resting blood pressure values (at least in a subset of animals), as they were not statistically different from uninjured CON or T10‐CON animals (Figure 2a–c).

### 3.3 Pressor responses to colorectal distension

**FIGURE 3:**
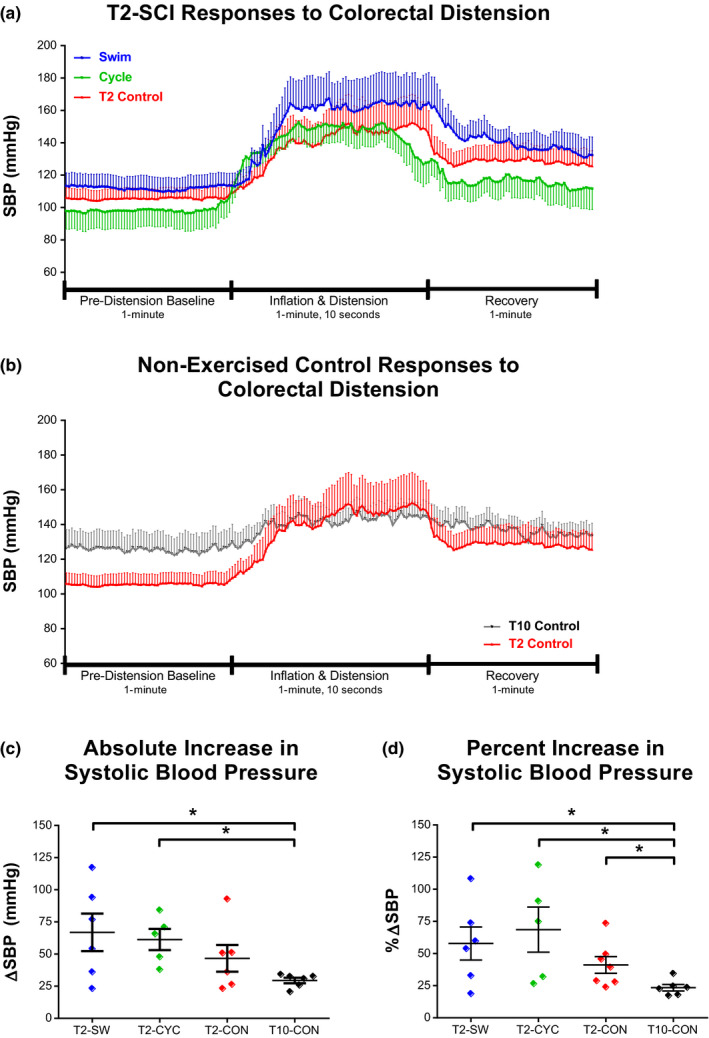
Exercise initiated early after T2 contusion did not attenuate the pressor response to colorectal distension. All T2‐SCI groups experienced significantly greater pressor responses to CRD than T10‐CON. (a) Time‐locked group average SBP data during pre‐distension baseline, inflation and distension, and recovery. Data are downsampled from 1000 Hz to one data point per second (a, b). The absolute increase in SBP was greater in exercised animals than in T2‐CON and T10‐CON (c). Also, SBP rise during CRD, as expressed as a percentage of baseline SBP, was significantly higher in all T2‐SCI groups, independent of exercise, as compared to T10‐CON (d). Data in c–d are represented as mean ± SEM and significance is set at *p ≤ .05

Responses to the CRD protocol from the T2‐SCI group are illustrated in Figure 3a. Similar responses illustrating T2‐CON versus T10‐CON animals are shown in Figure 3b. Pressor responses to CRD were observed in all contusion groups, irrespective of lesion level. However, the responses were much greater in T2‐SCI animals than in animals with T10 contusions. T10‐CON animals showed an absolute increase in SBP of less than 30 mmHg in response to CRD, whereas the three T2 groups (SW, CYC, and CON) exhibited responses of 67, 61, and 46 mmHg, respectively (Figure 3c: T2‐SW vs. T10‐CON, *p* = .037; T2‐CYC vs. T10‐CON, *p* = .006). In addition, the absolute change in DBP and MAP from rest was also significantly greater in T2‐SW and T2‐CYC compared to T10‐CON (DBP and MAP data not shown, all comparisons *p* ≤ .05). Neither active forelimb swimming nor passive hindlimb cycling exercise attenuated the pressor response to CRD as there were no differences between T2‐SCI group. Finally, when expressed as a percentage SBP increase from baseline, all T2‐SCI groups exhibited significantly greater pressor responses to CRD than T10‐CON animals (Figure 3d: T2‐SW vs. T10‐CON, *p* = .037; T2‐CYC vs. T10‐CON, *p* = .018; T2‐CON vs. T10‐CON, *p* = .015).

### 3.4 Recovery of CV function relates to terminal locomotor performance following T2 SCI

**FIGURE 4:**
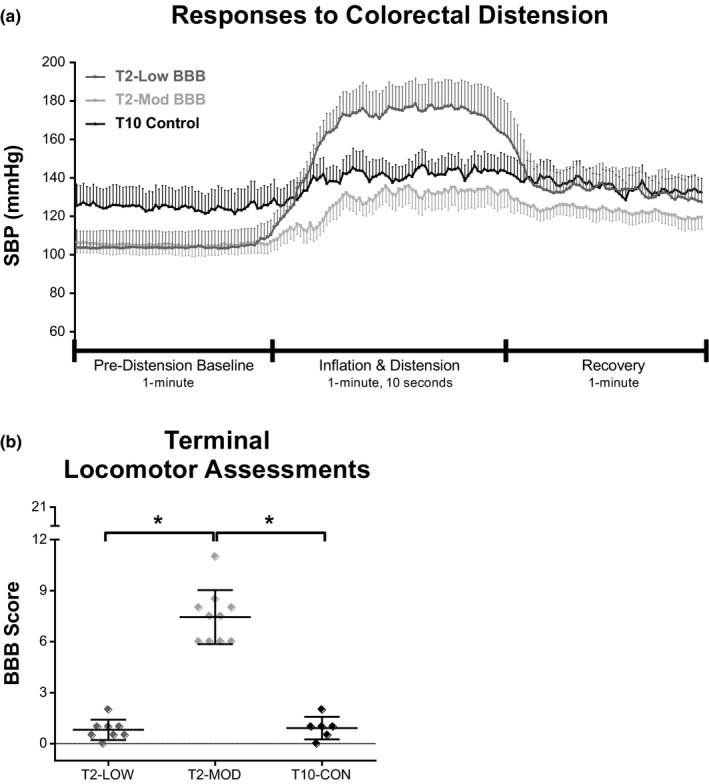
Pressor responses to colorectal distension are correlated with the recovery of locomotor function following severe T2 contusion. Animals with T2‐SCI were re‐classified according to terminal (week 5) performance on the BBB scale. Rodents with poor locomotor recovery (T2‐LOW) had significantly higher pressor responses to colorectal distension than T2‐MOD and T10‐CON. (a) Time‐locked group average SBP data during pre‐distension baseline, inflation and distension, and recovery. Data are downsampled from 1000 Hz to one data point per second (a). (b) Terminal BBB scores of T2‐SCI animals categorized into LOW and MOD hindlimb performance. Data are represented as mean ± SEM and significance is set at *p ≤ .05

Pressor responses varied considerably between T2‐SCI animals, irrespective of experimental condition. Therefore, responses to CRD were re‐analyzed based on terminal hindlimb function as assessed by the BBB Open Field Locomotor Scale. Using cluster analyses, all T2‐SCI animals were reclassified into T2‐LOW and T2‐MOD groups based on BBB scores less than 6 or 6 and greater, respectively. T10‐CON animals remained in their original cohort (i.e., were not reclassified based on BBB) and group comparisons were executed as previously described. Using this criteria, no T2‐LOW scored above 3 while the scores for the T2‐MOD animals ranged from 6 to 11, with the majority displaying sweeping of one or both hindlimbs without weight support (Figure 4b). As described earlier, T10‐CON animals had very little hindlimb function at the terminal time point (BBB scores ranged from 0 to 2) and scores were not different from the T2‐LOW animals. It is important to note that this reclassification of T2 animals was not equally distributed across the original groups––as discussed below, the majority of the T2‐LOW animals originated from the T2‐CYC group.

**FIGURE 5:**
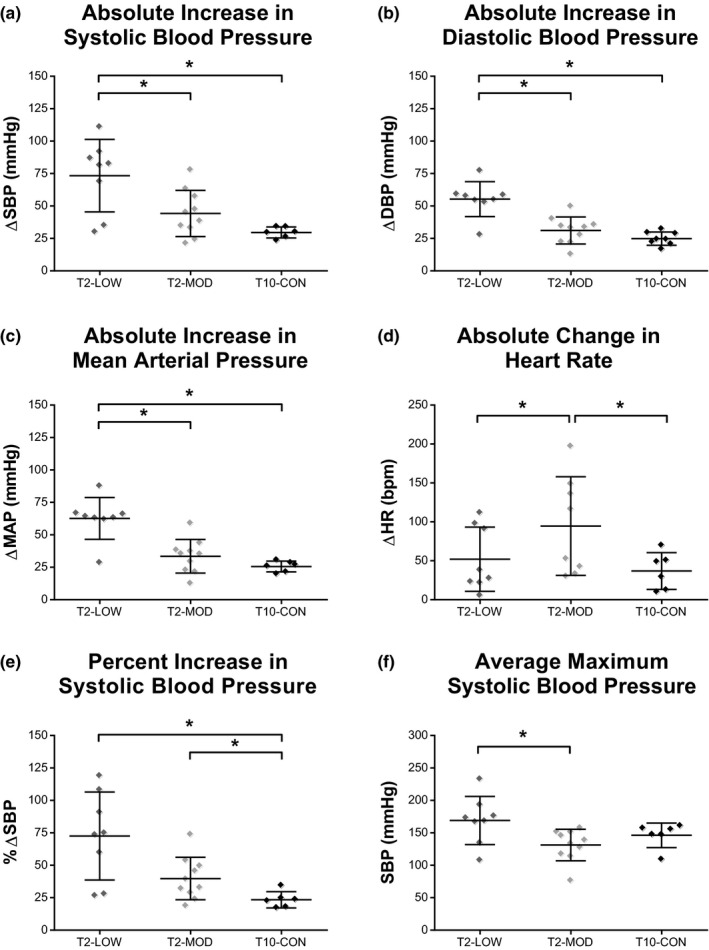
Severity of autonomic dysreflexia is greater in T2‐SCI rodents with limited hindlimb recovery. Pressor responses to colorectal distension were significantly greater in T2‐LOW. The absolute change in SBP (a), DBP (b), and MAP (c) was significantly greater in T2‐LOW than in T2‐MOD and T10‐CON. The maximum SBP during CRD was also greater in T2‐LOW versus T2‐MOD (f). Heart rate responses to the distension protocol were significantly greater in T2‐MOD compared to all other groups (d). Rodents with T10 contusion injuries did not experience significant pressor responses to CRD, as expressed as a percentage of baseline SBP, in comparison to both T2‐SCI groups (e). Significance is set at *p ≤ .05

Similar to the earlier analysis, T10 animals had significant resting tachycardia compared to the T2‐LOW and T2‐MOD groups (data not shown; T10‐CON vs. T2‐LOW, *p* = .039; T10‐CON vs. T2‐MOD, *p* = .009). Interestingly, this new stratification of T2‐injured animals revealed that rodents with minimal locomotor recovery developed the greatest degree of hemodynamic dysfunction. T2‐LOW animals displayed an exaggerated pressor response to CRD, where the absolute increase in SBP was higher than both T2‐MOD and T10‐CON groups (Figure 5a: T2‐LOW vs. T2‐MOD, *p* = .026; T2‐LOW vs. T10‐CON, *p* = .007). The absolute increase in DBP and MAP was also significantly higher in T2 animals with low BBB scores as compared to T2‐MOD and T10‐CON animals (Figure 5b,c, respectively; DBP: T2‐LOW vs. T2‐MOD, *p* = .003; T2‐LOW vs. T10‐CON, *p* = .005; MAP: T2‐LOW vs. T2‐MOD, *p* = .003; T2‐LOW vs. T10‐CON, *p* = .005). As before, SBP change as a percentage of pre‐distension baseline measures were significantly higher in both T2 groups as compared to the T10‐CON group (Figure 5e: T2‐LOW vs. T10‐CON, *p* = .005; T2‐MOD vs. T10‐CON, *p* = .023). There was also a trend for T2‐LOW animals to have higher percentage SBP responses to CRD than T2‐MOD (Figure 5e: T2‐LOW vs. T2‐MOD, *p* = .051). The average maximum SBP value reached during CRD was significantly higher in T2‐LOW as compared to T2‐MOD animals (Figure 5f; T2‐LOW vs. T2‐MOD, *p* = .021).

### 3.5 Recovery of hemodynamic stability following CRD

**FIGURE 6:**
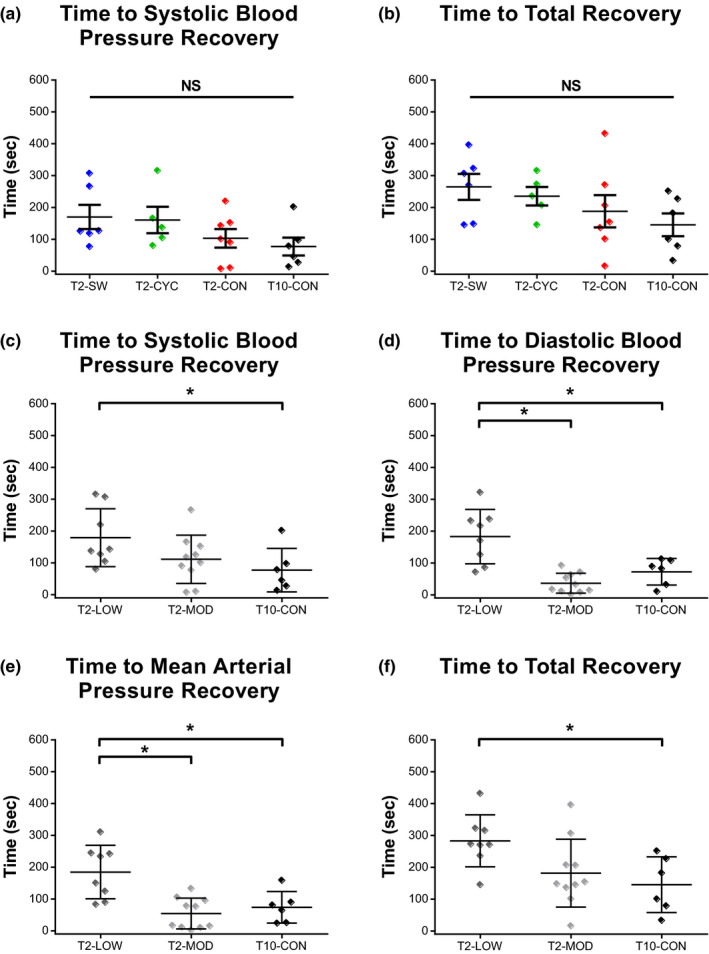
Rodents with more severe pressor responses to colorectal distension require longer recovery periods to reach resting hemodynamic parameters. The time needed for animals to reestablish pre‐distension hemodynamic parameters was calculated in seconds. T2‐LOW had significantly longer time to SBP (a), DBP (b), MAP (c), and total (d) recovery than T10‐CON animals. T2‐LOW also had significantly greater DBP and MAP recovery times than T2‐MOD. No differences were noted between T2‐MOD and T10‐CON. No differences were noted in any of the recovery variables when animals were analyzed in their original experimental groups (e, f). Original experimental group data are in e, f and are represented as mean ± SEM. Significance is set at *p ≤ .05

Pressor and heart rate responses to CRD were monitored for at least 10 min following deflation of the catheter balloon. The time needed, in seconds, for each animal to return to pre‐distension hemodynamic parameters (SBP, DBP, MAP, and HR) was calculated and compared across groups. No differences were noted when the original experimental groups were compared (Figure 6e,f: T2‐SW, T2‐CYC, T2‐CON, and T10‐CON). However, when terminal locomotor ability was used to stratify T2‐SCI animals, group differences revealed that time to recover hemodynamic stability was significantly longer for animals with greater CRD pressor responses. T2‐LOW had significantly longer recovery times than T10‐CON (Figure 6a–d; SBP, *p* = .02; DBP, *p* = .028; MAP, *p* = .02; and total recovery, *p* = .01), and T2‐MOD (Figure 6b,c; DBP, *p* = .001; MAP, *p* = .003).

## 4 DISCUSSION

### 4.1 Summary of findings

The present study shows that severe T2 contusive SCI results in episodes of unstable blood pressure regulation and severely disrupted CV homeostasis. Similar to studies using complete spinal transection, rodents with severe contusion exhibited robust pressor responses (AD) during experimentally induced colorectal distension. Dysregulation of CV homeostasis after SCI was not influenced by the early exercise training modalities employed, as neither swimming nor PHLC attenuated the pressor responses to CRD. Disordered CV control and severity of AD following T2 contusion appeared instead to be related to spontaneous functional recovery as assessed by the BBB scale.

### 4.2 Passive hindlimb cycling rehabilitation

Passive cycling is an attractive rehabilitation option over functional electrical stimulation (FES) cycling because it is simple and is not hampered by the various contraindications and side effects of electrical stimulation (Ashley et al., 1993). Passive hindlimb cycling has been examined extensively in the preclinical SCI literature as a rehabilitation modality for not only locomotor impairments, but also for CV dysfunction and neuropathic pain. Previous work by West and colleagues illustrated many CV benefits of acute PHLC rehabilitation following complete T3 transection, including reduced AD severity and nociceptor fiber sprouting in lumbar circuitry (West, Crawford, et al., 2014, 2015). In the current study, PHLC failed to reduce pressor responses to CRD following incomplete T2 contusion. The lack of improvement may be due to differences in the duration of daily training bouts (30 vs. 60 min). However, we find this unlikely given that only 15 min of PHLC training has been shown to augment levels of neurotrophic factors important for adaptive neuronal plasticity in the lumbar spinal cord, which in turn contributes to sensory and motor reflex recovery following complete SCI (Cote et al., 2011). Alternatively, the failure of PHLC to attenuate the severity of AD in the current study may be related to the level of locomotor recovery our rats experienced. Many animals in the T2‐CYC group developed at least extensive movement of one hindlimb joint. Given this ability, the passive nature of the cycling was, on occasion, contested by what appeared to be volitional or reflexive hindlimb kicking. Therefore, benefits gained through repetitive and predictable cyclic patterns of afferent activation may have been reduced.

### 4.3 Active swimming exercise

Active full‐body aerobic exercise has traditionally been preferred over passive rehabilitation for its effects on CV and respiratory systems (Ballaz et al., 2008; DeVeau et al., 2017; Hellsten & Nyberg, 2015; West et al., 2015c). However, paralysis of the trunk and lower limbs in combination with a blunted hemodynamic response to exercise makes this type of rehabilitation difficult or impossible for those within the SCI community (Van Loan et al., 1987). Active exercise is therefore limited to strategies involving the upper extremities. This is problematic, however, as there is general consensus that there is insufficient upper limb muscle mass to reap significant cardio‐metabolic benefits from arm ergometry alone (Davis et al., 1987; Gates et al., 2002). Unlike arm ergometry, swimming involves a significant need for trunk stability/orientation to ensure that the rodent swims in the appropriate direction during each lap. Previous studies in our lab showed that 4 min of swim exercise significantly challenges the CV system following incomplete T3 contusion in female rats (Harman et al., 2018). The current results suggest that intensive swimming exercise rehabilitation may tend to normalize resting hemodynamic parameters in animals with severe T2 contusions. However, pressor responses to CRD were not attenuated following this protocol, suggesting that a forelimb exercise alone, even when intense, is insufficient to alter or prevent the maladaptive processes leading to extreme pressor responses to CRD. Furthermore, this suggests that exercise paradigms lacking lower extremity involvement that fail to deliver predictable afferent stimulation to the lumbosacral spinal cord are unlikely to elicit favorable plasticity, or prevent unfavorable plasticity in central circuitry important for the development and maintenance of AD.

### 4.4 Relationships between motor and cardiovascular performance

The intensity of pressor responses to CRD varied considerably in animals with T2 contusions. In an effort to better understand the effects of incomplete injuries on the development and severity of AD, data from T2‐SCI animals were re‐analyzed based on terminal BBB scores. This analysis suggests a potential relationship between hindlimb locomotor capacity and pressor responses to experimentally induced CRD. Previous studies have reported a significant correlation between spared white matter at the lesion epicenter and hindlimb locomotor outcomes following a high thoracic impact of this severity (Basso et al., 1996). Our results show that animals with severe T2 contusions that developed very limited hindlimb motor recovery (LOW BBB group) experienced exacerbated pressor responses to CRD and longer recovery times that were independent of exercise rehabilitation. We speculate that this is likely due to the loss of sympathetic preganglionic neurons at the injury epicenter and/or reduced supraspinal input onto those neurons and reduced CV stimulus in the wake of low locomotor performance. Although not assessed, the lack of plantar paw placement and the accompanying afferent input to the lumbar spinal cord may have contributed to inappropriate sprouting and maladaptive plasticity in lumbosacral circuitry, further exacerbating pressor responses to CRD. In contrast, animals with greater hindlimb function (MOD BBB group) had CV responses that were not significantly different from the T10‐SCI group in which the pressor responses to CRD are considered to be within normal limits. In this group, animals presented with, at the very least, extensive movement of two hindlimb joints, and many animals displayed sweeping motion of the hindlimbs with/without plantar paw placement. The improvement in CV function noted in the T2‐MOD group may be due to the preservation of more descending sympatho‐excitatory and sympatho‐inhibitory pathways onto the sympathetic preganglionic neurons at the injury site, but may also reflect the influence of increased in‐cage hindlimb activity on maladaptive and adaptive plasticity in the lumbosacral circuitry. These data are in agreement with other studies demonstrating a strong correlation between injury severity, functional recovery, and the resulting CV control at rest and in response to provocation (Squair et al., 2016; Weaver et al., 2001; West et al., 2013). It is important to note that clinical studies have found both agreement and disagreement between the degree of neurological recovery and the severity of CV dysfunction. For instance, work by West and colleagues has demonstrated that some individuals considered neurologically motor and sensory “complete” (i.e., AIS A) according to the international standards for the classification of individuals with SCI, exhibit a “normal” CV response to exercise as well as an absence of blood pressure dysregulation (West et al., 2014b). Equally, it has been shown that some individuals with neurologically motor incomplete injuries (i.e., AIS C‐D) still exhibit CV and hemodynamic instability, though anecdotally this appears less common (Currie & Krassioukov, 2015; West, Wong, et al., 2014). Our present results do not refute these clinically observations, but rather lend support to the notion that relatively little spinal cord sparing (i.e., enough to produce a BBB score of 6–11 but not more) can partially mitigate or improve overt CV and hemodynamic dysfunction (Squair et al., 2016, 2018).

The results of this study also highlight the impact of lesion level on the recovery/maintenance of CV function following SCI. After injury, CV dysfunction can be attributed, in part, to the complete or partial loss of supraspinal control over preganglionic sympathetic neurons that provided excitatory drive to the heart, the splanchnic region, and upper body vascular beds (Furlan et al., 2003; Teasell et al., 2000). Importantly, the sympathetic innervation of the heart is supplied by fibers emerging from the T1–T5 spinal segments (Hou & Rabchevsky, 2014). Damage to this region of the spinal cord can disrupt homeostatic maintenance of the CV system due to the loss of facilitation and/or the lack of inhibition within the sympathetic nervous system (Teasell et al., 2000). As such, individuals with high level SCI are more susceptible to abnormalities in CV control, whereas patients with lesions at lower levels are less affected. We show that even with little to no hindlimb recovery, rodents with severe T10 contusion exhibit resting blood pressure levels that are within normal ranges, presumably due to the preservation of cardiac sympathetic preganglionic fibers. Furthermore, while there have been instances of AD in individuals with lesions as low as T8 or T10, this type of profound dysfunction is rare in patients with low thoracic and lumbar SCI (Gimovsky et al., 1985). Similar to what has been noted in the clinic, T10‐CON animals in this study had only modest increases in blood pressure following CRD provocation. These results suggest that, even in the wake of limited hindlimb locomotor capacity, animals with low thoracic injuries retain sufficient supraspinal control over the heart and upper body vasculature such that they are able to respond appropriately to baroreceptor‐mediated reflexes, maintain CV homeostasis, and thus limit clinically significant hemodynamic manifestations of CV system dysfunction.

### 4.5 Exercise training, plasticity, and cardiovascular outcomes

The development of therapeutic interventions to reduce the incidence of CVD in SCI patients continues to be a priority for clinicians and scientists alike. Chronic implementation of various rehabilitation strategies, such as FES cycling, have proven to be beneficial in improving vascular function (Ditor et al., 2005; Tordi et al., 2009) and aerobic capacity (DiCarlo et al., 1983; Groot et al., 2003) in the SCI community. However, to the best of our knowledge, there have been no studies that have sought to resolve the burden or severity of AD in patients with high thoracic or cervical injuries through acute exercise intervention. This is troublesome given the vast majority of these individuals experience frequent bouts of AD that effect everyday living and quality of life (Elliott & Krassioukov, 2006; Lindan et al., 1980).

The beneficial effects of exercise training after SCI have been noted numerous times in both clinical and preclinical settings. Therapeutic interventions such as wheelchair and arm ergometry have been shown to produce positive changes that include increased aerobic fitness, improved blood lipid profiles, and increased muscle mass and strength in chronic SCI patients (DiCarlo et al., 1983). Yet, the decline in physical fitness following injury is progressive and the length of time a patient remains immobile substantially impacts CV structure and function (Thijssen et al., 2006, 2012). Early implementation of exercise paradigms is ideal, as it would take advantage of inherent acute spinal plasticity and prevent maladaptive remodeling of both central and peripheral structures. However, the timing of exercise initiation and the intensity of exercise programs are both critical factors because starting “too early” may lead to physiological conditions, such as increased CGRP^+^ sprouting and extravasation of blood‐borne elements at the injury epicenter, that negatively impact recovery efforts (Laird et al., 2009; Smith et al., 2009). Furthermore, given the substantial spontaneous recovery of hindlimb function and trunk stability exhibited by rodents with SCI, exercise paradigms must surpass the in‐cage activity and potential for innate retraining they exhibit. Indeed, data from this study supports the notion that hindlimb recovery and related in‐cage locomotion may contribute to reduced pressor responses to CRD. Improvements in over‐ground locomotion and plantar placement of the hindlimbs will result in increased volume and pressure loading of the heart and appropriate afferent stimulation of the lumbar circuitry, thereby creating a new steady state for CV mechanics and exercise training. While it is important to note that the T2 model presented in this study is not usual for investigating locomotor recovery, exercise rehabilitation, and CV function after SCI, the variability inherent in this model presents some important aspects to consider when applying exercise prescription to the general SCI population. The use of an incomplete injury, even of this severity, allows for more variability in spontaneous recovery, which, in turn is influenced by adaptive/maladaptive plasticity occurring in the lumbar spinal cord. If harnessed appropriately, that variability may ultimately lead to improvements in CV outcomes and reduced incidence of CVD. This is also true for the clinical population, keeping in mind that the injury may differentially impact motor, sensory, and autonomic circuitry leading to unique conditions of injury completeness. The heterogeneity of human injuries and the level of immobility following injury will undoubtedly influence the potential impact of rehabilitation strategies and the resulting CV improvements.

In summary, this novel study examined the CV effects of two different modalities of exercise rehabilitation following severe but incomplete thoracic SCI. Our data are consistent with previous published work in that high thoracic SCI results in episodes of unstable blood pressure regulation and severely disrupted CV homeostasis. Unfortunately, we were not able to show overt improvements in AD severity following short periods (3.5 weeks) of either form of exercise training. Instead, our data suggests that spontaneous locomotor recovery (as assessed by the BBB) may help to mitigate the development and severity of AD in this model. If so, future studies that aim to change the trajectory of CV decline following incomplete SCI using early training programs should consider the impact of spontaneous locomotor recovery and post‐SCI plasticity and adapt the exercise prescription accordingly.

## CONFLICT OF INTEREST

The authors declare no conflict of interest, financial, or otherwise.

## AUTHOR CONTRIBUTIONS

Author contributions: K.A.H., K.M.D., J.W.S., A.V.K., C.R.W., and D.S.K.M. conceived and designed the research project; K.A.H., K.M.D., J.W.S., and A.V.K. performed the experiments; K.A.H. analyzed the data; K.A.H., C.W., and D.S.K.M. interpreted the results of the experiments; K.A.H. prepared figures and drafted the manuscript; K.A.H., K.M.D., J.W.S., A.V.K., C.R.W., and D.S.K.M. edited and revised the manuscript; K.A.H. and D.S.K.M. approved the final version of manuscript.
